# H19X-encoded miR-424(322)/-503 cluster: emerging roles in cell differentiation, proliferation, plasticity and metabolism

**DOI:** 10.1007/s00018-018-2971-0

**Published:** 2018-11-24

**Authors:** Fan Wang, Rui Liang, Neha Tandon, Elizabeth R. Matthews, Shreesti Shrestha, Jiao Yang, Benjamin Soibam, Jin Yang, Yu Liu

**Affiliations:** 1 Department of Oncology, The First Affiliated Hospital of Xian Jiaotong University, Xi’an, 710061 Shaanxi China; 2 Department of Biology and Biochemistry, University of Houston, Houston, TX 77204 USA; 3 Computer Science and Engineering Technology, University of Houston-Downtown, Houston, TX 77002 USA

**Keywords:** H19X, miR-424, miR-322, miR-503, Tumor suppressor gene, EMT, Hypoxia

## Abstract

miR-424(322)/-503 are mammal-specific members of the extended miR-15/107 microRNA family. They form a co-expression network with the imprinted lncRNA H19 in tetrapods. miR-424(322)/-503 regulate fundamental cellular processes including cell cycle, epithelial-to-mesenchymal transition, hypoxia and other stress response. They control tissue differentiation (cardiomyocyte, skeletal muscle, monocyte) and remodeling (mammary gland involution), and paradoxically participate in tumor initiation and progression. Expression of miR-424(322)/-503 is governed by unique mechanisms involving sex hormones. Here, we summarize current literature and provide a primer for future endeavors.

## Introduction

MicroRNAs (miRNAs) are small non-coding RNAs 18–24 nucleotides (nt) in length that regulate posttranscriptional gene expression [1]. miRNA genes are transcribed by RNA polymerase II to produce pri-miRNA, which is cleaved by Drosha to give rise to hairpin-structured pre-miRNA, ~ 60–100 nt in length. Exportin transports pre-miRNA into the cytoplasm, where it is processed by Dicer to produce a ~ 22 nt double-stranded intermediate comprising the mature miRNA strand and its complementary strand. The mature miRNA is loaded into the RNA-induced silencing complex (RISC) where it binds to the 3′ UTR of target mRNAs by partial sequence complement in the “seed” region, causing degradation of the mRNA transcript or inhibition of its translation.

The miR-15/107 family of microRNAs shares the “AGCAGC” sequence within the “seed” region, starting at either the first or the second nucleotide from the 5′ end [2]. They are critical regulators of cell division, apoptosis, stress response and metabolism, and involved in cancer, cardiovascular and neurodegenerative disorders. miR-424 (ortholog of rodent miR-322) and miR-503 are mammal-specific members of the miR-15/107 family. They are encoded as one cluster by H19X, located in human Xq26.3 [3]. Their expression is more dynamic and tissue restrictive than other miR-15/107 family members. miR-424(322)/-503 regulate fundamental processes such as cell cycle, epithelial-to-mesenchymal transition and hypoxia, drive tissue differentiation and remodeling, and paradoxically participate in tumor initiation and progression. Here, we summarize a decade of literature and provide a primer for future investigation concerning miR-424(322)/-503.

## miR-424(322)/-503 are unique members of the miR-15/107 miRNA family

### The miR-15/107 family of miRNAs

**Fig. 1:**
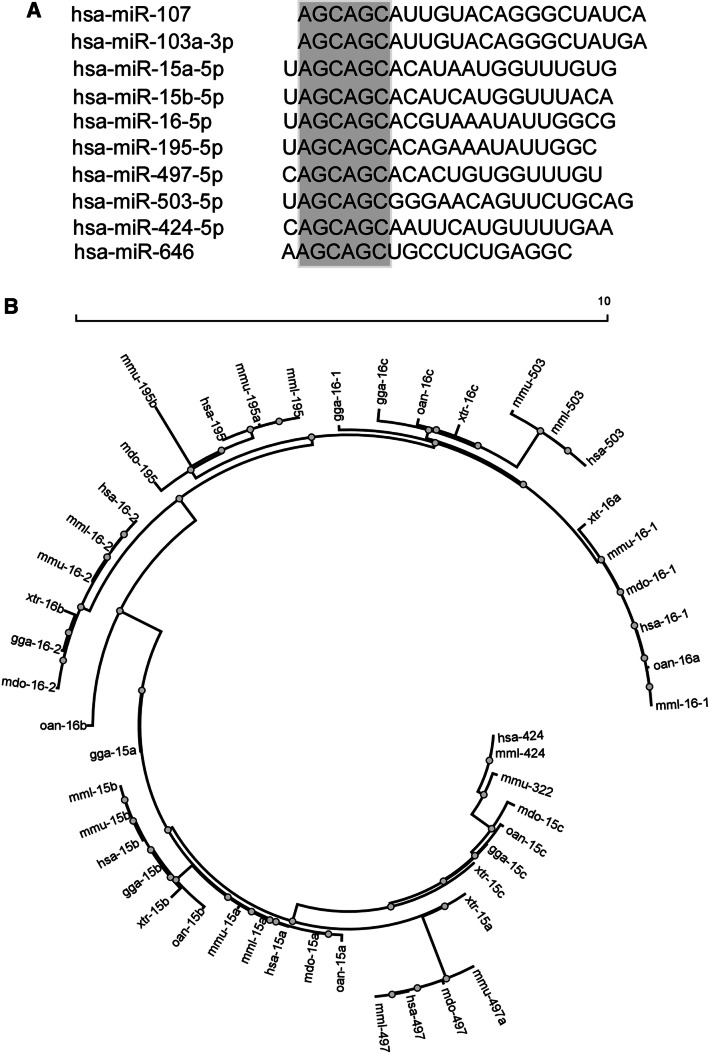
Members of the miR-15/107 microRNA family. **a** 10 microRNAs sharing “AGCAGC” within the “seed” region. **b** A phylogenetic tree of miR-15/107 family members from *Homo sapiens* (*hsa*), *Monodelphis domestica* (*mdo*), *Macaca mulatta* (*mml*), *Mus musculus* (*mmu*), *Ornithorhynchus anatinus* (*oan*), *Gallus gallus* (*gga*) and *Xenopus tropicalis* (*xtr*). miR-103 and -107 are the least closely related, and omitted in the plot. miR-424(322) is most closely related to miR-15c in *mdo*, *oan*, *gga* and *xtr*, while miR-503 is most closely related to miR-16c in *gga*, *oan* and *xtr*

The miR-15/107 family includes ten miRNAs based on the presence of “AGCAGC” in the “seed” region situated at positions 2–7 from the 5′ end of mature microRNAs [2]. However, there is no consensus regarding the criteria of miRNA family classification; distinct classifications were proposed for these ten miRNAs [4–9]. We have adopted the classification by Finnerty et al. [2], but this is likely to change with new functional data accumulated and new miRNAs identified (Fig. 1a).

miR-15/107 family members are only expressed in chordates, with several being mammal specific (miR-195, -497, -503, -424 and -646) [2]. Genes of miR-15/107 family members are genomically associated with protein-coding genes or lncRNAs. They also show conserved tandem organization: miR-15 with miR-16, miR-424(322) with miR-503, and miR-497 with miR-195. To explore the evolutionary relation of miR-424(322)/-503 to others, we have updated a phylogenetic tree originally built by Necsulea et al. [3], using “stem-loop” pre-miRNA sequences in *Homo sapiens*, *Monodelphis domestica*, *Macaca mulatta*, *Mus musculus*, *Ornithorhynchus anatinus*, *Gallus gallus* and *Xenopus tropicalis* extracted from miRBase. miR-424 (together with miR-322 and miR-15c) and miR-503 (together with miR-16c) represent two distinct subfamilies related to miR-15 and miR-16, respectively (Fig. 1b).

Supporting a common phylogenetic origin, miR-15/107 family members have similar expression patterns and functions. miR-15a, -15b, -16, -322 and -503 are dynamically upregulated during serum starvation and contact inhibition, with miR-503 showing the highest fold change [10]. miR-15a, -15b, -16 and -497 are essential for the switch from expansion to differentiation in precursor B lymphocytes [11]. On the other side, loss of either miR-15a/-16-1 or miR-15b/-16-2 by genomic deletion causes B cell chronic lymphocytic leukemia [12–14]. miR-15/-16 family members also drive NK cell maturation, by targeting Myb [15]. miR-15/107 family members inhibit cell proliferation in many tissue types. They are broadly upregulated after birth and cause cardiomyocyte mitotic arrest in rodents [16, 17]. Additionally, miR-15/107 family members respond to cellular stresses such as hypoxia, ischemia, ultraviolet, environmental toxin, etc., and induce adaptive changes in angiogenesis and cellular metabolism [18–23]. As will be detailed in the rest of this essay, the function of miR-424(322)/-503 overlaps with other family members, but is under unique temporal and spatial regulations and works in distinct processes.

### Association with the imprinted paradigm lncRNA, H19

Necsulea et al. surveyed eight organs in 11 tetrapod species for the expression profiles of ncRNAs [3]. They have identified approximately 400 lncRNA genes that are at least 300 million years old. These lncRNAs evolve rapidly in terms of sequence and expression levels, but conserve tissue specificity. One evolutionarily conserved co-expression network is predicted to regulate placenta development. This network comprises H19 and the lncRNA that encodes the miR-424(322)/-503 cluster, which was hence named H19X.

H19 is best known as the imprinting paradigm [24–29]: except under rare pathological conditions, the H19 gene is only expressed from the maternal allele, while the adjacent IGF2 gene is only expressed from the paternal allele. Imprinting provides an important mechanism of gene dose control, allowing expression from only one allele, while the other is epigenetically silenced. Many imprinted genes are involved in placenta development; H19 regulates placenta growth in late gestation, via its “spinoff” miR-675 [30]. Overexpression of H19 causes embryonic and perinatal lethality [31]. H19 is quickly downregulated in most tissues except skeletal muscles after birth [32, 33].

H19X resembles H19 in several ways. First, H19X may be imprinted (see following section). Second, miR-424 is downregulated by hypoxia in trophoblasts, and higher miR-424 levels are associated with fetal growth restriction, indicating a role in placenta growth regulation [34, 35]. Third, expression of H19X is striated muscle restricted during embryogenesis. In addition to these suggestive evidences, it will be important to know if H19 and H19X are mutually regulatory, and if they cooperate with or compensate for each other in genetic models. Addressing these questions may provide insights into the intricate mechanisms of gene dose control and coordination in embryonic development.

### Structure of the H19X locus

**Fig. 2:**
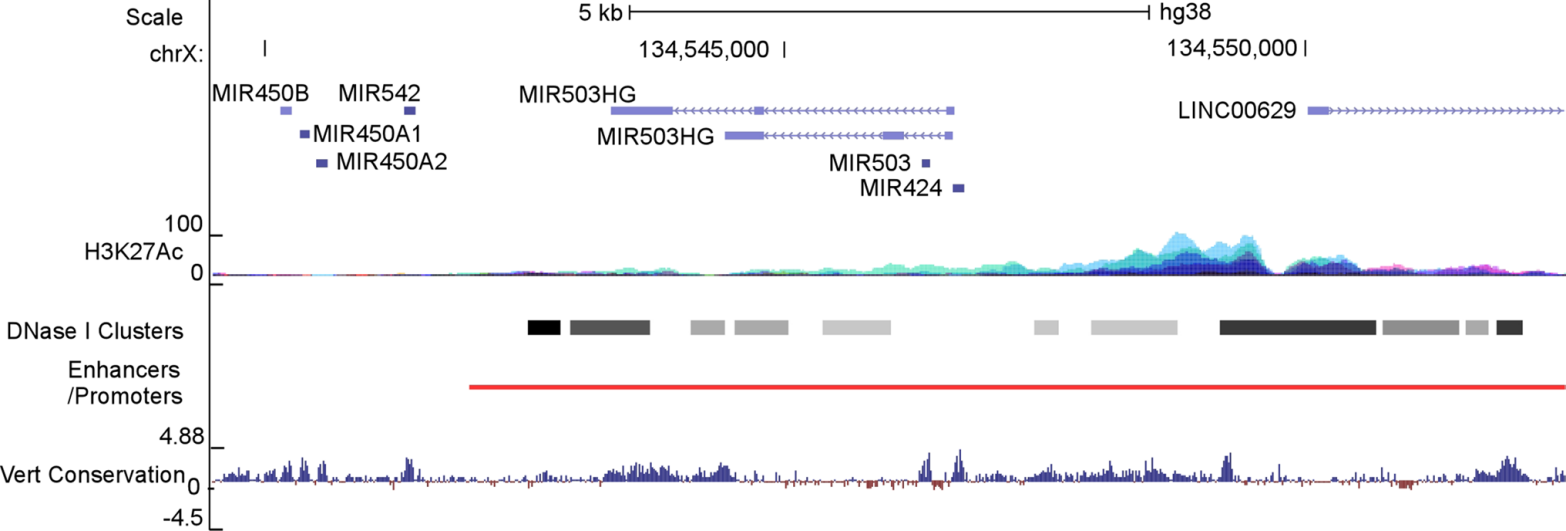
Schematic diagram of the H19X locus. miR-424, -503, -542, -450b, -450a1 and -450a1, and at least one lncRNA, miR503HG, are encoded in the H19X locus. H19X spans approximately 7 k nucleotides on the minus strand of Xq26.3. It is unknown if other adjacent ncRNAs, such as linc00629, are related to H19X. In the upstream regulatory regions as well as gene bodies, active epigenetic marks including H3K27Ac and DNAse I-hypersensitive clusters were identified by the ENCODE project; a high-confidence enhancer/promoter cluster was identified by the GeneHancer project. Information obtained from UCSC Genome Browser

The human H19X locus encodes seven non-coding RNAs, including a number of microRNAs (miR-424, 503, 542, 450-1, 450-2, and 450b) and a long non-coding RNA (miR503HG), spanning a region of ~ 7 kb pairs on Xq26.3 (Fig. 2). The microRNAs are highly conserved in mammals. The lncRNA has similar expression patterns as miR-424(322)/-503, agreeing with being the host gene of the miRNA cluster. The ENCODE project has identified clustered H3K27Ac and DNAse I signals, while the GeneHancer project has identified a high-confidence cluster of regulatory elements in the upstream regulatory region and gene bodies of miR-424 [36], miR-503 and miR503HG, suggesting that the H19X locus is actively transcribed and intricately regulated.

In human and mouse genomes, H19X is situated between PLAC1 and HPRT on the 5′ and 3′ ends, respectively. PLAC1 is a placenta-specific protein whose expression is restricted in the trophoblast lineage. PLAC1 is paternally imprinted, and deficiency of it causes placentomegaly [37]. In marsupials, the downstream gene of H19X is RNA-on-the-silent X (Rsx), an Xist-like lncRNA that drives X-inactivation [38]. Collectively, the genomic location and indication in placenta growth suggest that H19X is probably imprinted.

The precursor sequence of the miR-424(322)/-503 cluster encodes both -5p and -3p mature miRNAs. According to expression levels, miR-322-5p, miR-424-5p and miR-503-5p are predominant over their -3p counterparts. The -3p miRNAs are not related to the miR-15/107 family. Throughout this manuscript, miR-322, miR-424 and miR-503 refer to -5p mature miRNAs. There are few studies about the function of -3p miRNAs encoded by the miR-424(322)/-503 cluster; how these -3p miRNAs contribute to described phenotypes is completely unknown.

## Roles in cell fate specification and differentiation

### Cardiomyocyte differentiation

**Fig. 3:**
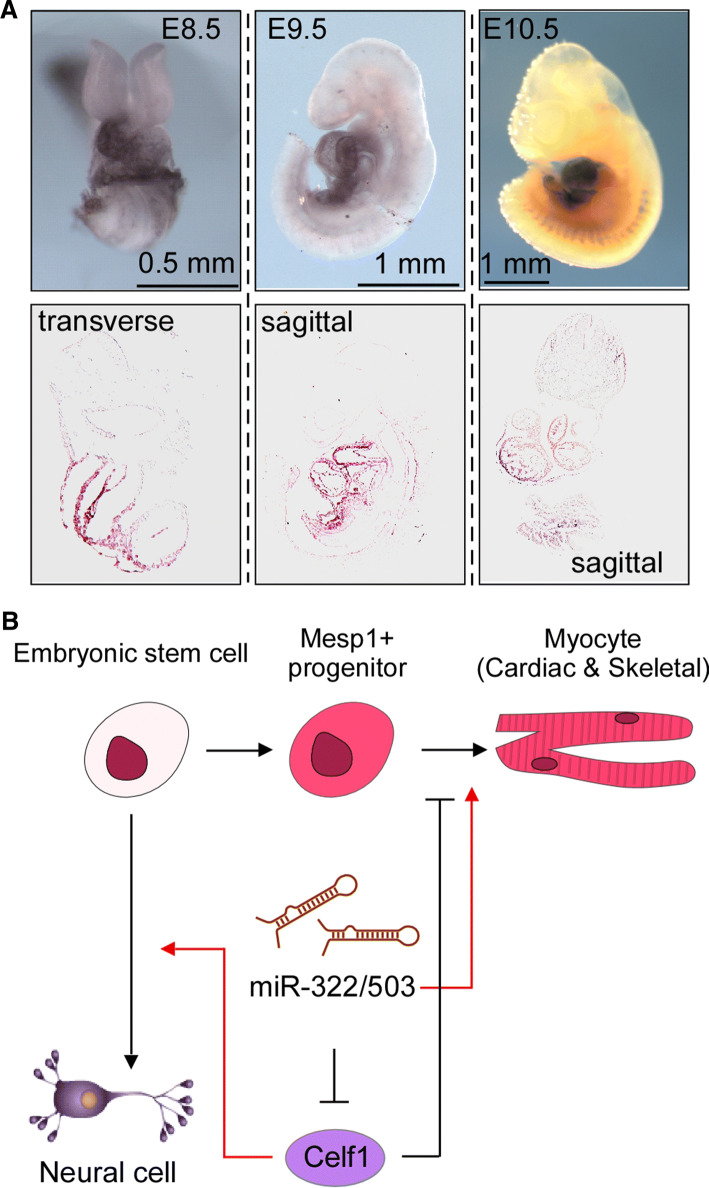
Role of miR-322/-503 in embryonic myocyte differentiation. **a** miR-322/-503 are specifically expressed in the embryonic hearts at E8.5, E9.5 and E10.5, and in the somites at E10.5. **b** A working model: miR-322/-503 target Celf1 and augment cardiac and skeletal muscle cell differentiation at the cost of neuroectoderm derivatives

In the embryo proper, expression from the H19X locus first appears in Mesp1-marked mesendoderm cells. Mesp1 is a bHLH factor that is transiently expressed at the onset of gastrulation [39, 40], marking a bipotent cardiac and skeletal muscle precursor population [41]. By surveying the transcriptome of this precursor population, we found that miR-322 and -503 were highly enriched, together with other H19X ncRNAs [42]. A knockin LacZ reporter demonstrated that expression from H19X is restricted to the developing heart, somites and skeletal muscles (Fig. 3a). miR-322/-503 augment the cardiomyocyte differentiation program while inhibiting neuroectoderm cell fates. Mechanistically, miR-322/-503 target Celf1 (note: only functionally and biochemically validated direct targets are included in this review), an RNA-binding protein that regulates RNA alternative splicing and decay and has a tight neuroectoderm association in embryos [42] (Fig. 3b). Mesendoderm formation and cardiac differentiation represent the first epithelial-to-mesenchymal transition (EMT) in life; as discussed later, regulating EMT is one of the chief mechanisms of miR-424(322)/-503 in carrying out their functions.

### Skeletal muscle differentiation

Agreeing with specific expression in skeletal muscle precursor cells, miR-322(424)/-503 promote skeletal muscle differentiation [43]. Skeletal muscle differentiation starts with a G1 phase cell cycle arrest through inhibition of CDK2 [44, 45]. miR-322(424)/-503 target Cdc25A, the phosphatase that removes inhibitory phosphorylation on CDK2 and causes cell cycle progression [43]. This effect of miR-322(424)/503 is implicated in inducing cell cycle quiescence during muscle differentiation.

Most miR-15/107 family members are broadly expressed [2]. As to whether some members have relative tissue specificity, such as whether miR-103/-107 are highest in the brain, and whether miR-15/-16 are higher in hematopoietic cells, discrepant results exist [46–50]. Striated muscle restriction appears to be unique for miR-322/-503. It is one place where H19 and H19X cross path. H19 is broadly expressed in embryos, but restricted in postnatal skeletal muscles [33, 43], whereas H19X shows early restriction which is lost after birth [42]. Whether their functions are related or even coordinated remain to be determined.

### Monocyte differentiation

The transcription factor PU.1 synergizes with miR-424 in driving transcriptional commitment in the differentiation from promyelocytic blasts to monocyte/macrophage lineages [51]. PU.1 transactivates miR-424; miR-424 then targets NFI-A, whose downregulation is required for the commitment of two myeloid-specific pathways (granulocyte and monocyte/macrophage). miR-424, therefore, has a high hierarchical position in monocyte/macrophage lineage differentiation. Other mechanisms of miR-424 in inducing monocyte differentiation have also been described. miR-155, miR-222, miR-424 and miR-503 are induced by phorbol myristate acetate which promotes terminal differentiation of acute myeloid leukemia cells blocked in a progenitor cell state; miR-155 arrests cells in G2 phase, miR-424/-503 arrest cells in G1 and miR-222 induces apoptosis [52]. Interestingly, miR-424/503 directly targets the anti-differentiative miR-9 [52]. In chronic myeloid leukemia, miR-424 suppresses proliferation by directly targeting the oncogenic BCR-ABL tyrosine kinase fusion gene [53].

One of the best characterized functions of miR-15/-16 is in hematopoiesis and leukemia [54, 55]. miR-15a/-16-1 are lost by 13q deletions in chronic lymphocytic leukemia, which leads to activation of miR-15/-16 target, BCL2 [56, 57]. There appears a trend where miR-15/-16 regulate lymphoid cell differentiation, whereas miR-424(322)/-503 contribute to myeloid cell differentiation [11, 51, 52]. These miRNAs are predicted to target overlapping sets of mRNAs; therefore, they are likely controlled by different lineage commitment transcription factors to carry out similar functions in the lymphoid and myeloid compartments, respectively.

## Roles in regulating proliferation and apoptosis, and as a tumor suppressor

### Molecular targets in cell division and apoptosis

miRNAs have been long known to modulate cell cycle. Members of the miR-15/107 family induce G1 arrest by targeting primary cell cycle regulators including CDK1, CDK2, CDK6, cyclin D1, cyclin D3 and cyclin E1 [5, 6, 58–60]. Together with other miR-15 family members, miR-424(322)/-503 are upregulated in G1-arrested cells, serum starvation, contact inhibition and cellular senescence, and the change in miR-322/-503 levels is a multitude higher than others [10, 61]. Moreover, miR-15/-16 regulate cell death by targeting BCL2, which is an important mechanism in chronic lymphocytic leukemia [56, 57]. Like miR-15/-16, miR-424(322)/-503 are pro-apoptotic. Regulatory functions in cell division and apoptosis underlay the involvement of miR-424(322)/-503 in many biological processes, such as differentiation, organ homeostasis and carcinogenesis. To this front, Llobet-Navas et al. exemplarily dissected how miR-424(322)/-503 regulate mammary gland involution and work as a breast cancer tumor suppressor gene [62, 63].

### Function in mammary gland involution and as a tumor suppressor

The mammary gland continuously undergoes tissue remodeling [64]. During pregnancy, secretory alveoli develop and form a dense lactiferous epithelial tree. During weaning, the alveoli and secretory duct structure collapses, known as involution. Llobet-Navas et al. described a cascade of molecular events in the involuting process [62]. Weaning activates the TGF-β signaling pathway, which upregulates the expression of the primary transcript of miR-424(322)/-503. Once processed, the mature miRNAs target important genes involved in cell division (Cdc25A) and survival or death decisions (Bcl-2 and Igf1r), resulting in significant reduction in the activity of the AKT and ERK1/2 pathways. These are conducive to inhibited growth and increased apoptosis of mammary epithelial cells. Ablation of the miR-424(322)/-503 gene leads to compromised regression of the secretory acini of the mammary gland. Further, ablation of miR-424(322)/-503 promotes breast tumorigenesis after pregnancy in animal models [63]. miR-424(322)/-503 is frequently lost in a subset of aggressive primary breast tumors which are chemoresistant, due to increased activity of BCL-2 and IGF1R. Other miR-15/107 family members are not among the most changed during mammary gland involution, supporting that regulating mammary epithelial cell growth is a unique function of miR-424(322)/-503.

A large corpus of evidence supports that miR-424(322)/-503 work as a tumor suppressor whose deletion or downregulation contributes to tumor initiation or aggressive behavior. We summarize the findings in Table 1, with apologies to colleagues whose works are not included due to space. We choose to highlight a few where multiple lines of evidences are available, with emphasis given to gynecological cancers.

**Table 1:** Tumor suppressor roles of miR-424(322)/503

miR	Cancer site	Pathology	Target	Refs.
miR-424-5p	Breast	Basal-like	DCLK1	[65]
miR-322/-503	Breast	NA	CDC25A, CCNE1, BCL-2, IGF1R	[62, 63]
miR-503	Breast	NA	PYK2	[66]
miR-424-5p	Cervix	Adenocarcinoma	KDM5B	[67]
miR-424	Cervix	Cervical epithelial carcinoma	CHK1	[68]
miR-503	Esophagus	ESCC	CCND1	[69]
miR-503	Esophagus	ESCC	HOXC13	[70]
miR-503	Esophagus	ESCC	PRKACA	[71]
miR-424	HSC	CML	BCR-ABL	[53]
miR-424-5P	Liver	HCC	ICAT	[72]
miR-503	Liver	HCC	FGF2, VEGF-A	[73]
miR-503	Liver	HCC	EIF4E	[74]
miR-503	Liver	HCC	Cyclin D3, E2F3	[75]
miR-503	Lung	NSCLC	PI3K p85, IKK-β	[76]
miR-424(322)	Ovary	Epithelial ovarian carcinoma	PD-L1, CD80	[77]
miR-424	Ovary	OCCC	DCLK1	[78]
miR-503	Prostate	NA	ZNF217	[79]
miR-424	Uterus	Endometrial carcinoma	E2F7	[80]
miR-503	Uterus	EEC	CCND1	[81]

*ESCC* esophageal squamous cell carcinoma, *CML* chronic myelogenous leukemia, *HCC* hepatocellular carcinoma, *NSCLC* non-small cell lung cancer, *OCCC* ovarian clear cell carcinoma, *EEC* endometrioid endometrial cancer

Several groups independently uncovered that miR-424 is downregulated in cervical cancers; lower miR-424 is correlated with poor prognostic clinicopathological parameters [67, 68, 82–85]. Xu et al. identified that miR-424 targets CHK1, which is inversely correlated to miR-424 levels [68]. Ectopic miR-424 enhanced apoptosis and blocked G1/S transition, and suppressed cell migration and invasion in cervical cancer cell lines. Intriguingly, this mechanism is indicated in human papillomavirus infection, a causative event of cervical and other anogenital cancers [83]. HPV E6 and E7 proteins suppress the levels of miR-424, while CHK1 is augmented. As a downstream effector kinase in the ATR DNA repair pathway, increased CHK1 contributes to viral genome amplification.

Lower miR-424 levels are also associated with epithelial ovarian cancers [78, 86]. Ectopic miR-424-5p arrests ovarian cancer cells in G0/G1 phase, via directly inhibiting cyclin E1 [86]. In ovarian clear cell carcinoma, a subtype of epithelial ovarian cancer associated with poor prognosis and chemoresistance, miR-424 targets doublecortin-like kinase 1 (DCLK1) which is associated with cancer stem cells in multiple cancers [78]. Moreover, miR-424(322) modulates the PD-L1/PD1 and CD80/CTLA4 immune checkpoint [77]. PD1 and CTLA4 are T cell-expressed immunomodulatory receptors, whereas PD-L1 (to PD1) and CD80 (to CTLA4) are binding partners present on tumor cells and macrophages, and dendritic cells, respectively. PD-L1/PD1 and CD80/CTLA4 interactions result in reduced CD8 + cytotoxic T-lymphocyte proliferation and survival, and ultimately immune tolerance [87]. In chemoresistant epithelial ovarian cancers, miR-424(322) directly targets PD-L1 and CD80. Lower miR-424(322) and higher PD-L1 correlate to chemoresistant phenotypes [77]. Restoration of miR-424(322) hence represents a new opportunity in increasing chemosensitivity in ovarian cancers.

## Regulation by hormones

The expression of miR-424(322)/-503 is significantly altered during several hormone-controlled processes. Endometriosis is a benign gynecological disease among women in reproductive age, characterized by the presence of endometrial glands and stroma in locations other than the uterine cavity. Several groups reported the downregulation of miR-424 or miR-503 in endometrial tissues vs. controls, or in ectopic vs. eutopic endometrial tissues [88–90]. There is an inverse correlation between miR-424 and the levels of VEGF-A [89]. Higher VEGF-A levels may be responsible for elevated angiogenic activity in endometriotic lesions. Ovary granulosa cells support the growth and maturation of follicles, and they undergo constant morphological and functional changes. miR-424/-503 are highly expressed in granulosa cells, with varying expression levels during the menstrual cycle [91–93]. miR-424/-503 regulate proliferation of granulosa cells, but how they affect follicle growth and maturation is unknown [91, 94].

Two groups have reported that expression of miR-424(322)/-503 is responsive to estrogen in MCF-7 breast epithelial cells. One group captured the temporal profiles of miRNAs and identified both miR-424 and miR-503 as among the most upregulated by E2 [95]. The other group captured the temporal profiles of mRNAs following E2 exposure and reversely predicted miRNAs that may be regulated. miR-424 was among the top findings and experimentally vetted [96]. Though these studies support the close association of miR-424 and -503 with estrogen, it is unknown if an estrogen receptor element is present on the upstream regulatory region and essential for H19X transcription. Efforts in this area will provide new insights into hormone-related disease mechanisms. Further, as miR-424(322)/-503 emerge as a critical regulator of muscle differentiation, growth and metabolism [42, 43, 97], their hormone link may help explain sex differences in muscle physiology and diseases.

## Roles in epithelial-to-mesenchymal transition and tumor progression

### Relation to the TGF-β pathway and epithelial-to-mesenchymal transition (EMT)

The transforming growth factor beta family comprises structurally related proteins that regulate cell proliferation, differentiation, apoptosis and other functions [98–100]. TGF-β proteins bind to TGF-β type II receptors, which phosphorylate the type I receptor on its serine residues, and the latter becomes activated. Next, type I receptors phosphorylate R-SMADs (SMAD2 and SMAD3 for TGF-βs), increasing their affinity to coSMAD (SMAD4). Finally, the RSMAD/coSMAD complex translocates to the nucleus where it functions to drive gene transcription. The TGF-β pathway is regulated at multiple levels; R-SMADs are regulated by inhibitory SMADs (I-SMADs) and proteasome-mediated degradation. There are two I-SMADs: SMAD6 competes with R-SMADs for SMAD4 binding, whereas SMAD7 competes with R-SMADs for binding to type I receptors. Both I-SMADs are downstream targets of TGF-β signaling, providing a negative feedback mechanism. E3 ubiquitin ligases SMURF1 and SMURF2 regulate the levels of R-SMADs via proteasome-mediated degradation.

Mounting evidences support that TGF-β signaling is one of the main drivers of miR-424(322)/-503 biogenesis [62, 101–104]. In mammary epithelial cells, activated TGF-β signaling leads to increased transcription of miR-424(322)/-503, through an upstream SMAD-binding site [62]. Gu et al. found that SMAD4 is required for miR-503 transactivation, through the same SMAD-binding site, during smooth muscle cell differentiation in mesenchymal stem cells [102]. Additionally, TGF-β upregulates miR-424 in glioblastoma, cardiac fibrosis, myofibroblast differentiation from lung epithelial cells and formation of cancer-associated fibroblasts (CAFs) [101, 103, 104].

**Fig. 4:**
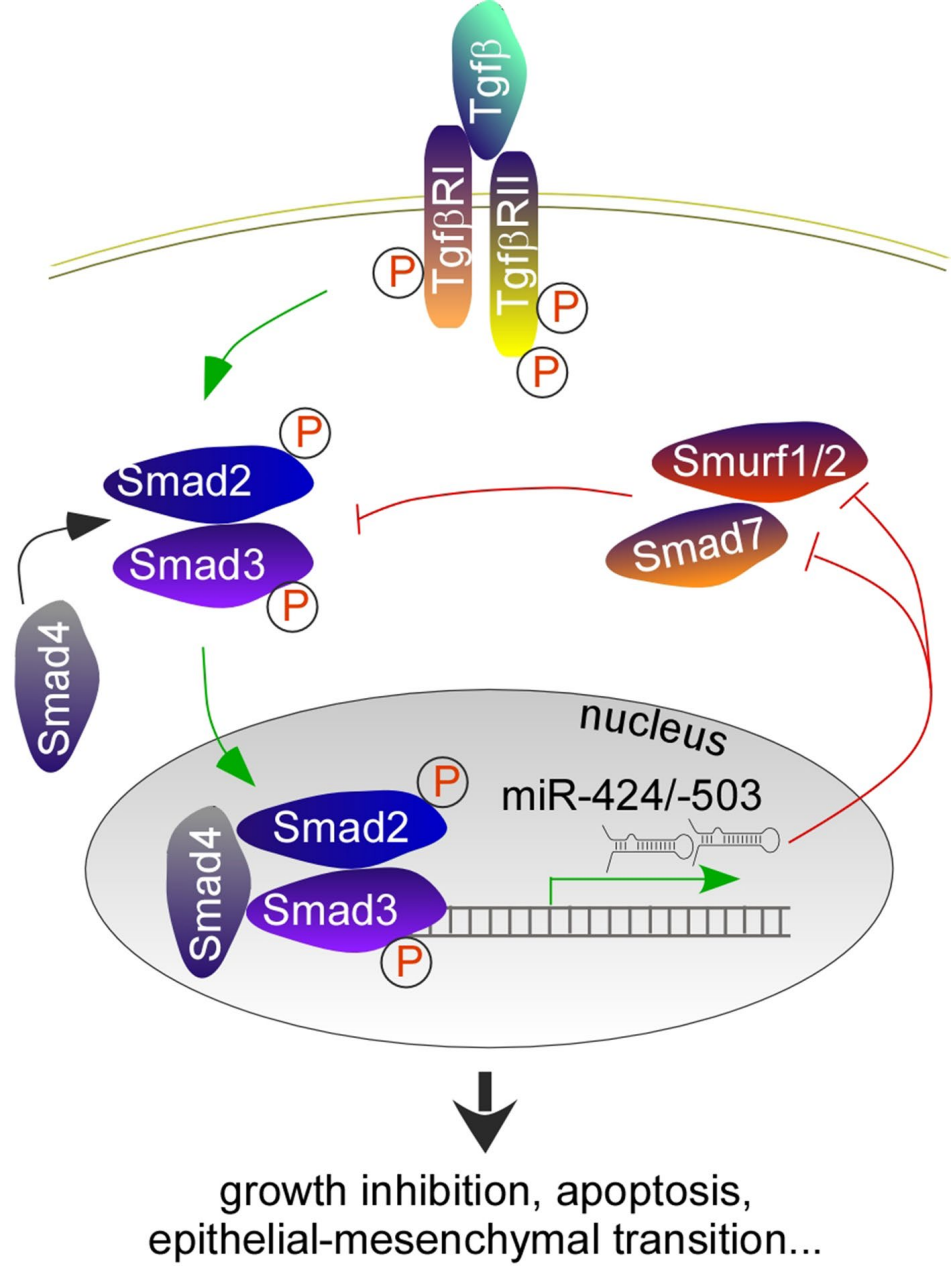
A feedforward mechanism in TGF-β signaling. miR-424(322)/-503 are direct downstream targets of the TGF-β/SMAD2/3 signaling pathway. They inhibit the expression of I-SMAD (SMAD7) and SMAD-specific E3 ubiquitin protein ligases (SMURF1/2), therefore effectively activate SMAD2/3 and amplify TGF-β signaling

In some processes, miR-424(322)/-503 provide a feedforward mechanism to amplify TGF-β signaling (Fig. 4). In distraction osteogenesis, a clinical strategy to promote bone formation, miR-503 is one of the most upregulated miRNAs. miR-503 targets SMURF1, and positively modulates TGF-β signaling which is active during the early stage of distraction osteogenesis [105]. During TGF-β-induced EMT in human lung epithelial cells and TGF-β-regulated intestinal epithelial homeostasis, miR-424(322)/-503 target SMURF2 and enhance TGF-β signaling [103, 106]. The inhibitory SMAD7 is also a target of miR-424, which relieves the negative effects of SMAD7 on R-SMADs and contributes to smooth muscle cell differentiation in mesenchymal stem cells [102].

miR-424(322)/-503 help carry out classic function of TGF-β, such as growth inhibition and EMT, by serving as an effector and feedforward regulator. Confoundingly, miR-424 may also negatively regulate TGF-β signaling by targeting TGFBR3 [107, 108]. Thus, miR-424(322)/-503 target selection may be specific for individual processes, but more likely, miR-424(322)/-503 may target a few TGF-β pathway components simultaneously; the net outcome is a balance among all the interactions. This agrees with the notion that miRNAs regulate a network of genes by fine-tuning their expression levels, and hence coordinating a biological process.

miR-424(322)/-503-mediated EMT contributes to tumor progression in some cancers. Drasin et al. described stage-dependent roles of miR-424 in breast cancer [107]: downregulated miR-424 leads to tumor initiation, while subsequent upregulation facilitates metastasis. Twist and Snai1, classic transcription factors of mesenchymal programming, drive miR-424 expression. Elevated miR-424 induces EMT and cancer stemness-associated genes, by selectively targeting TGFBR3. In metastases, miR-424 is downregulated, which facilitates MET [107]. Completion of the EMT–MET axis allows metastatic tumor outgrowth at the new site. The importance of miR-424(322)/-503 in regulating EMT and cellular plasticity has also been demonstrated in colorectal cancer, prostate cancer and tongue squamous cell carcinoma [108–110].

### Other mechanisms contributing to tumor progression

Suppressor of cytokine signaling (SOCS) factors are negative regulators of the JAK/STAT pathway. Lowered SOCS expression leads to higher JAK/STAT activity, which is associated with many cancers [111]. In oral squamous cell carcinoma, SOCS2 is downregulated and inversely correlated with the level of miR-424-5p. There exists a signal-amplifying loop in which IL-8 drives activity of STAT5, STAT5 induces the expression of miR-424-5p, and miR-424-5p enhances the activity of STAT5 by inhibiting SOCS2. This signal loop mediates IL-8-induced cell migration and invasion [112].

Dallavalle et al. reported another mechanism of miR-424 in activating STAT proteins. In prostate tumors, miR-424 is upregulated due to lower expression of ESE3/EHF, which binds to the promoter of miR-424 and represses its transcription. E3 ubiquitin ligase COP1, an miR-424 target, is downregulated. Consequently, several oncogenic transcription factors including STAT3 evade proteasome-mediated degradation and become activated [113].

miR-424-5p facilitates gastric cancer cell proliferation and invasion by targeting LATS1, a core component of the Hippo pathway [114]. Circular RNA_LARP4 neutralizes the activity of miR-424-5p by serving as a molecular sponge. This is a rare demonstration of a posttranscriptional mechanism in regulating miRNA accessibility.

In summary, the role of miR-424(322)/-503 in cancer is highly contextual. While a tumor suppressive role has been established in breast cancers, oncogenic functions are suggested in glioblastoma and melanoma [63, 101, 115, 116]. The elegant works in breast cancer demonstrate the dynamic roles of miR-424(322)/-503 through the initiation and progression of the disease, exemplifying the complexity of gene regulation exerted by miRNAs. Potential redundant or cooperative roles of other miR-15/107 family members pose additional challenges in understanding the role of miR-424(322)/-503 in cancer. Despite the confounding issues, miR-424(322)/-503 has emerged as critical regulators of a variety of cancer hallmarks. Additional genetic evidences, as well as better appreciation of the dynamic interactions of miR-424(322)/-503 with its target network may bring about new therapeutic opportunities.

## Roles in stress response

Expression of miR-15/107 family members is responsive to a variety of cellular stresses, including UV damage, environmental toxin, hypoxia and ischemic injury [18–23]. Among miR-15/107 family members, miR-424(322)/-503 show the highest responsiveness under stress. [10]. Here we summarize the underlying mechanisms that miR-424(322)/-503 use to alleviate damages and help adaptation during cellular stresses.

### Hypoxia and ischemia

**Fig. 5:**
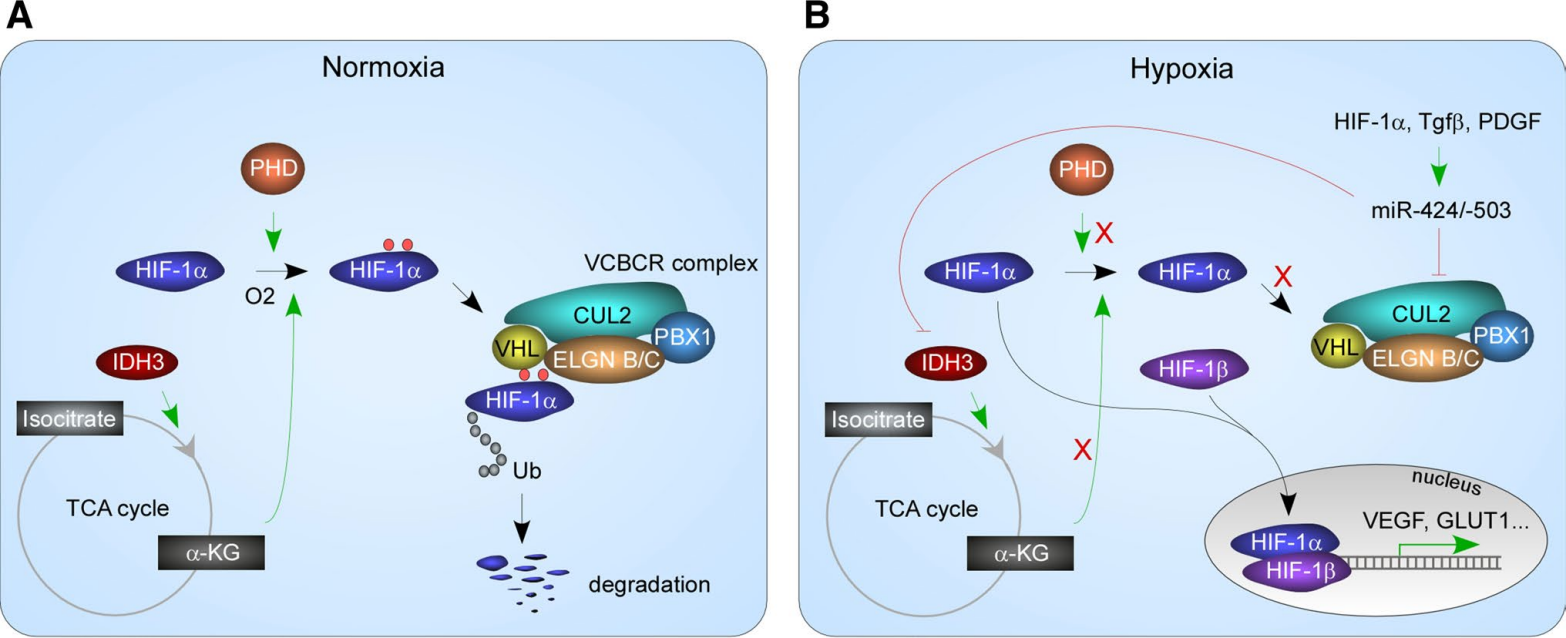
miR-424/-503 mediated mechanisms in HIF-1α stabilization. In normoxia, HIF-1α is hydroxylated by PHD, using O2 and α-KG. Hydroxylated HIF-1α is recruited to the VCBCR complex in which CUL2 serves as the scaffold. HIF-1α is degraded by the proteasome system. In hypoxia, miR-424(322)/-503 are activated by factors including HIF-1α. They target the TCA cycle enzyme, IDH3, and drop the levels of α-KG. They also target CUL2 and inhibit the formation of the VCBCR complex. Collectively, miR-424(322)/-503 stabilize HIF-1α and enhance cellular response to hypoxia

Reduced availability of oxygen can occur in physiological conditions such as wound healing and physical exertion as well as pathological situations such as stroke and myocardial ischemia. A complex adaptation system centered around hypoxia-inducible factor (HIF) is responsible for restoring oxygen and nutrient homeostasis [117] (Fig. 5a). Under normoxia, HIF-1α is maintained at low levels due to active proteasome-mediated degradation. The degradation process starts with hydroxylation on two proline residues, P402 and P564. Hydroxylated HIF-1α is recognized by von Hippel–Lindau (VHL) protein, which brings hydroxylated HIF-1α to the VCBCR (VHL, elongin C, elongin B, cullin 2, and RBX1) E3 ubiquitin ligase complex. Poly-ubiquitination and proteasome-mediated degradation ensue. In hypoxia, HIF-1α becomes stabilized and forms a heterodimer with HIF-1β. The dimer migrates into the nucleus where it binds to hypoxia response elements and transactivates genes regulating metabolism, angiogenesis and erythropoiesis.

miR-424 and miR-210 are the most upregulated miRNAs in hypoxic vascular endothelial cells which sit at the frontline of responding to hypoxia. Ectopic miR-424 stabilizes both HIF-1α and HIF-2α, through targeting cullin 2 (CUL2), the scaffold protein for the E3 ubiquitin ligase complex [118] (Fig. 5b). Accordingly, miR-424 overexpression promotes in vitro angiogenesis and neovascularization in mice. Induction of miR-424 in hypoxia is through a C/EBPα-RUNX-1/PU.1 cascade, in which C/EBPα in cooperation with RUNX-1 transactivates PU.1 expression, and PU.1 transactivates miR-424 transcription through a PU.1-binding site [118]. Hypoxia induces miR-424 in a myocardial infarction mouse model as well as a hind limb ischemia mouse model, demonstrating the response is widespread in multiple tissue types [118].

Hypoxia induces miR-424 in cancer cells as well. Zhang et al. reported that in melanoma and colon cancer cell lines, hypoxia transactivates miR-424 expression via a hypoxia response element present on the promoter of miR-424. Increased miR-424 renders resistance to apoptosis-inducing drugs doxorubicin and etoposide [119].

Cerebral ischemia induces an acute increase in miR-424 levels in the peri-infarct cortex in a middle cerebral artery occlusion/reperfusion mouse model. Ectopic miR-424 reduces neuronal cell apoptosis and infarct volume, accompanied by increased activity of MnSOD [120]. In neuronal culture, H_2_O_2_ upregulates miR-424 expression, similar to ischemia or hypoxia exposure. Thus, it appears that the dynamically reactive miR-424 provides an acute means of reducing oxidative stress [120].

### Endoplasmic reticulum stress

Deregulation of normal endoplasmic reticulum (ER) function leads to a conserved cellular response, unfolded protein response (UPR) [121]. It can trigger cell death if ER stress is prolonged. There are three ER transmembrane proteins serving as sensors of unfolded protein accumulation in the ER lumen: PERK1, IRE1, and ATF6. They represent three branches of signaling pathways in restoring homeostasis. Thapsigargin and tunicamycin, drugs causing accumulation of unfolded protein in the ER lumen, downregulate miR-424(322)/-503 in a PERK1-dependent fashion. miR-424 modulates the activity of two branches of UPR: it directly binds to the 3′-UTR of ATF6 transcripts and inhibits translation, whereas it regulates the activity of “regulated IRE1 dependent decay” (RIDD) on the IRE1 branch. Together, PERK-induced downregulation of miR-424(322)/-503 optimizes the activity of IRE1 and ATF6 during ER stress, hence serving as a node to coordinate UPR [122].

## Roles in metabolism

Consistent with a role in adapting cells to changed environment, miR-424 has been shown to regulate major metabolic switches. TGF-β or PDGF-induced CAF formation is accompanied by metabolic switch from oxidative phosphorylation to aerobic glycolysis [104]; miR-424 plays a critical role in the process. TGF-β upregulates the expression of miR-424, and miR-424 directly targets isocitrate dehydrogenase 3a, an enzyme catalyzing the conversion from isocitrate to α-ketoglutarate (α-KG) in the tricarboxylic acid (TCA) cycle. Upregulated miR-424 causes a drop in α-KG, which contributes to the stability of HIF-1α. Proteasome-mediated HIF-1α degradation requires HIF-1α hydroxylation on two proline residues, P402 and P564; the activity of the responsible enzyme, proline hydroxylase, requires oxygen and α-KG. Structural analogs of α-KG, such as succinate and fumarate, inhibit proline hydroxylase activity [123–125]. Increased miR-424 effectively drops the ratio between α-KG and succinate/fumarate, and reduces HIF-1α degradation [104]. This is another mechanism that miR-424 uses to stabilize HIF-1α (Fig. 5). Stabilized HIF-1α transactivates genes involved in glycolysis.

Diabetic levels of glucose significantly drive down miR-424 expression in breast cancer cells [126]. Lowered miR-424 levels lead to higher expression of its target gene, CDC42. CDC42 induces the expression of transcription factor PRDM14, which is associated with poor prognosis in breast cancer patients [126]. Wang et al. reported decreased miR-150, miR-146a and miR-424 in peripheral blood mononuclear cells from type I diabetic patients, and the decrease is associated with ongoing autoimmunity of pancreatic islet [127]. However, it is not clear how miR-424 is suppressed under hyperglycemic conditions, and how this change contributes to adaptive or pathological alterations. Research in this area is not yet sufficient to build a unitary framework, but miR-424(322)/-503 apparently influences metabolic pathways, which serve as bridges linking many processes discussed so far.

## As biomarkers

Using circulating miRNAs as biomarkers has gained tremendous research interests [128, 129]. Many cell types, such as reticulocyte, dendritic cell, B cell, T cell, mast cell, epithelial cell, as well as tumor cell, release miRNAs. They are incorporated into exosomes/extracellular vesicles (EVs) and transferred to body fluids, such as plasma, urine and saliva. Exosomes/EVs carry and deliver mRNAs and miRNAs into recipient cells and exert profound physiological and pathological functions [130, 131]. Meantime, these circulating RNAs constitute a new category of non-invasive disease markers.

Circulating miR-15/-16 show correlation with several cancer types, including glioma, esophageal adenocarcinoma, cervical cancer and breast cancer [132–135]. They exhibit prognostic value in melanoma and acute heart failure [136, 137]. miR-424(322)/-503, especially miR-424, often constitute miRNA signatures with high predictive power for disease outcomes. Bye et al. assayed 179 miRs in the serum of 112 healthy participants who either suffered from fatal AMI within 10 years or remained healthy. They established a model for predicting future AMI consisting of miR-106a-5p, miR-424-5p, let-7g-5p, miR-144-3p and miR-660-5p, with 74.1% and 81.8% correct classification for men and women, respectively [138]. de Andrade et al. showed in 39 ALS patients/39 controls that miR-424 and miR-206 were higher in patient plasma and the baseline levels were associated with clinical deterioration [139]. Two groups independently demonstrated the prognostic value of miR-424 in non-small cell lung cancers. One model includes four miRNAs (miR-200c, miR-424, miR-29c and miR-124), whereas the other includes six miRNAs (miR-29a, miR-542-5p, miR-502-3p, miR-376a, miR-500a, miR-424), each holding prognostic value for overall survival [140, 141]. A 3-miRNA signature (miR-199a, miR-29c and miR-424) was found to distinguish breast cancer patients from controls [142]. We have summarized recent reports about miR-424(322)/-503 as circulating biomarkers in a variety of diseases (Table 2). Though there are many caveats regarding using circulating miRNA as biomarkers, it is clear that miR-424 is one of the best candidates that may be vetted in larger cohorts.

**Table 2:** Detection of miR-424(322)/-503 as biomarkers in body fluids

miRNA	Disease or physiological status	Sample	Expression change	Method	Refs.
miR-424	Breast cancer	Serum	+	SdM-RT-PCR	[142]
miR-424	Advanced NSCLC	Blood	+	miRNA microarray	[141]
miR-424	FXTAS	Blood	+	miRNA microarray and sequencing	[143]
miR-424	ALS	Skeletal muscle, plasma	+	miRNA microarray	[139]
miR-503	Vertebral fractures	Serum	−	qRT-PCR	[144]
miR-503	Postmenopausal osteoporosis	Blood	−	miRNA microarray	[145]
miR-424	PH	Serum	+	qRT-PCR	[146]
miR-424-5p	Type 1 diabetes	Serum	+	qRT-PCR	[147]
miR-424-5p	DVT	Plasma	+	qRT-PCR array	[148]
miR-424-5p	AMI	Serum	+	qRT-PCR array	[138]
miR-424-5p	Heart failure	Plasma	−	qRT-PCR array	[149]
miR-503	CAD	Plasma	−	qRT-PCR	[150]
miR-424-3p, miR-424-5p	Aerobic exercise	Serum	+	qRT-PCR	[151]

*sdM-RT-PCR* serum-direct multiplex detection assay based on RT-PCR, *NSCLC* non-small cell lung cancer, *FXTAS* fragile X-associated tremor/ataxia syndrome, *ALS* amyotrophic lateral sclerosis, *PH* pulmonary hypertension, *DVT* deep-vein thrombosis, *AMI* acute myocardial infarction, *CAD* coronary artery disease

A major challenge is that the source of the circulating miRNAs is often unknown. It is critical to distinguish whether they are from a primary lesion such as cancer, or a secondary and reactive source, such as lymphocytes or muscles. Technical issues include the lack of a housekeeping circulating RNA control to normalize among individuals and different diseases, and variations introduced by different extraction and quantification methods. The importance of addressing these challenges cannot be overestimated; it likely constitutes a major milestone in diagnostics.

## Conclusions and future perspectives

Over a decade of research has accumulated a large body of knowledge related to the miR-424(322)/-503 cluster. Individually or together, these miRNAs are involved in placenta, heart and skeletal muscle development during embryogenesis. They regulate core cellular processes including cell cycle control and EMT. They are the most dynamic in responding to a range of cellular stresses, including hypoxia and ischemia, and help restore homeostasis. Hormonal regulation over the biogenesis of these miRNAs and their involvement in physiological and pathological processes of female reproduction organs prompt an important question, are they one of the deciding factors of sex differences? The identification of miR-424(322)/-503 as biomarkers in many human diseases, especially the frequent detection in plasma samples, poses exciting clinical opportunities. However, this is still a new research subject; there remain many challenges awaiting exploration.

Many important hypotheses are not validated. The connection to H19 has not been supported by genetic evidence in animal models. Does miR-424(322)/-503 overexpression stall placental development, like in H19 transgenic mouse? Is the H19X locus paternally imprinted? Genetic ablation of H19 or miR-424(322)/-503 causes very mild systemic phenotypes: H19 KO animal has mild overgrowth [29], whereas miR-424(322)/-503 KO animal has mild white fat accumulation [62]. Will double-knockout animal display more pronounced phenotypes related to organism growth or homeostasis? Answering these questions would provide important insights into ncRNA regulatory mechanisms of growth control.

It remains difficult to rank the importance of molecular targets of miRNAs in a biological process. Most studies rely on computation programs or transcriptome survey to predict miRNA targets, and select one or two for additional investigation. Such strategies have intrinsic weaknesses: miRNAs are known to target many genes simultaneously, and mainly through affecting protein abundance, not mRNA abundance. With the advancement of technologies such as reverse phase protein array (RPPA), we may include protein arrays into the toolbox of miRNA target identification. This note is especially important for miR-424(322)/-503, as they are highly dynamic and their identified targets sometimes occupy opposite sides of signaling pathways.

Developing therapeutic interfering strategies require understanding of the redundancy and coordination between miR-424(322)/-503 and other miR-15/107 family miRNAs. It is a challenging task to inhibit the activities of miR-424(322)/-503, because other miR-15 family members may be parallel or compensatory. Thus, developing strategies that target all the miRNAs sharing the same seed sequence would provide conclusive evidence for the function of miR-424(322)/-503 in important processes, and also form the base for therapy development.

Finally, building connections among the currently separated processes is critical. For instance, under cellular stress, cell proliferation, plasticity and metabolism may be well orchestrated, and miR-424(322)/-503 may coordinate the expression of a network of genes and help cells adapt and regain homeostasis. Systemic dissection of these processes, especially at protein and organism levels, would likely yield important insights that are currently unavailable.

## References

[1] Ambros V (2004). The functions of animal microRNAs. Nature.

[2] Finnerty JR, Wang WX, Hebert SS, Wilfred BR, Mao G, Nelson PT (2010). The miR-15/107 group of microRNA genes: evolutionary biology, cellular functions, and roles in human diseases. J Mol Biol.

[3] Necsulea A, Soumillon M, Warnefors M, Liechti A, Daish T, Zeller U, Baker JC, Grutzner F, Kaessmann H (2014). The evolution of lncRNA repertoires and expression patterns in tetrapods. Nature.

[4] Choong ML, Yang HH, McNiece I (2007). MicroRNA expression profiling during human cord blood-derived CD34 cell erythropoiesis. Exp Hematol.

[5] Linsley PS, Schelter J, Burchard J, Kibukawa M, Martin MM, Bartz SR, Johnson JM, Cummins JM, Raymond CK, Dai H, Chau N, Cleary M, Jackson AL, Carleton M, Lim L (2007). Transcripts targeted by the microRNA-16 family cooperatively regulate cell cycle progression. Mol Cell Biol.

[6] Liu Q, Fu H, Sun F, Zhang H, Tie Y, Zhu J, Xing R, Sun Z, Zheng X (2008). miR-16 family induces cell cycle arrest by regulating multiple cell cycle genes. Nucleic Acids Res.

[7] Xia L, Zhang D, Du R, Pan Y, Zhao L, Sun S, Hong L, Liu J, Fan D (2008). miR-15b and miR-16 modulate multidrug resistance by targeting BCL2 in human gastric cancer cells. Int J Cancer.

[8] Small EM, Frost RJ, Olson EN (2010). MicroRNAs add a new dimension to cardiovascular disease. Circulation.

[9] Beveridge NJ, Gardiner E, Carroll AP, Tooney PA, Cairns MJ (2010). Schizophrenia is associated with an increase in cortical microRNA biogenesis. Mol Psychiatry.

[10] Rissland OS, Hong SJ, Bartel DP (2011). MicroRNA destabilization enables dynamic regulation of the miR-16 family in response to cell-cycle changes. Mol Cell.

[11] Lindner SE, Lohmuller M, Kotkamp B, Schuler F, Knust Z, Villunger A, Herzog S (2017). The miR-15 family reinforces the transition from proliferation to differentiation in pre-B cells. EMBO Rep.

[12] Kasar S, Underbayev C, Hassan M, Ilev I, Degheidy H, Bauer S, Marti G, Lutz C, Raveche E, Batish M (2016). Alterations in the mir-15a/16-1 loci impairs its processing and augments B-1 expansion in de novo mouse model of chronic lymphocytic leukemia (CLL). PLoS One.

[13] Lovat F, Fassan M, Gasparini P, Rizzotto L, Cascione L, Pizzi M, Vicentini C, Balatti V, Palmieri D, Costinean S, Croce CM (2015). miR-15b/16-2 deletion promotes B-cell malignancies. Proc Natl Acad Sci USA.

[14] Klein U, Lia M, Crespo M, Siegel R, Shen Q, Mo T, Ambesi-Impiombato A, Califano A, Migliazza A, Bhagat G, Dalla-Favera R (2010). The DLEU2/miR-15a/16-1 cluster controls B cell proliferation and its deletion leads to chronic lymphocytic leukemia. Cancer Cell.

[15] Sullivan RP, Leong JW, Schneider SE, Ireland AR, Berrien-Elliott MM, Singh A, Schappe T, Jewell BA, Sexl V, Fehniger TA (2015). MicroRNA-15/16 antagonizes Myb to control NK cell maturation. J Immunol.

[16] Porrello ER, Johnson BA, Aurora AB, Simpson E, Nam YJ, Matkovich SJ, Dorn GW, van Rooij E, Olson EN (2011). MiR-15 family regulates postnatal mitotic arrest of cardiomyocytes. Circ Res.

[17] Porrello ER, Mahmoud AI, Simpson E, Johnson BA, Grinsfelder D, Canseco D, Mammen PP, Rothermel BA, Olson EN, Sadek HA (2013). Regulation of neonatal and adult mammalian heart regeneration by the miR-15 family. Proc Natl Acad Sci USA.

[18] Pothof J, Verkaik NS, van Ijcken W, Wiemer EA, Ta VT, van der Horst GT, Jaspers NG, van Gent DC, Hoeijmakers JH, Persengiev SP (2009). MicroRNA-mediated gene silencing modulates the UV-induced DNA-damage response. EMBO J.

[19] Kulshreshtha R, Ferracin M, Wojcik SE, Garzon R, Alder H, Agosto-Perez FJ, Davuluri R, Liu CG, Croce CM, Negrini M, Calin GA, Ivan M (2007). A microRNA signature of hypoxia. Mol Cell Biol.

[20] Donker RB, Mouillet JF, Nelson DM, Sadovsky Y (2007). The expression of Argonaute2 and related microRNA biogenesis proteins in normal and hypoxic trophoblasts. Mol Hum Reprod.

[21] Yin C, Salloum FN, Kukreja RC (2009). A novel role of microRNA in late preconditioning: upregulation of endothelial nitric oxide synthase and heat shock protein 70. Circ Res.

[22] Yin KJ, Deng Z, Hamblin M, Xiang Y, Huang H, Zhang J, Jiang X, Wang Y, Chen YE (2010). Peroxisome proliferator-activated receptor delta regulation of miR-15a in ischemia-induced cerebral vascular endothelial injury. J Neurosci.

[23] Rahman M, Lovat F, Romano G, Calore F, Acunzo M, Bell EH, Nana-Sinkam P (2014). miR-15b/16-2 regulates factors that promote p53 phosphorylation and augments the DNA damage response following radiation in the lung. J Biol Chem.

[24] Bartolomei MS, Zemel S, Tilghman SM (1991). Parental imprinting of the mouse H19 gene. Nature.

[25] Gabory A, Jammes H, Dandolo L (2010). The H19 locus: role of an imprinted non-coding RNA in growth and development. BioEssays.

[26] Yoo-Warren H, Pachnis V, Ingram RS, Tilghman SM (1988). Two regulatory domains flank the mouse H19 gene. Mol Cell Biol.

[27] Tilghman SM, Bartolomei MS, Webber AL, Brunkow ME, Saam J, Leighton PA, Pfeifer K, Zemel S (1993). Parental imprinting of the H19 and Igf2 genes in the mouse. Cold Spring Harb Symp Quant Biol.

[28] Bartolomei MS, Webber AL, Brunkow ME, Tilghman SM (1993). Epigenetic mechanisms underlying the imprinting of the mouse H19 gene. Genes Dev.

[29] Ripoche MA, Kress C, Poirier F, Dandolo L (1997). Deletion of the H19 transcription unit reveals the existence of a putative imprinting control element. Genes Dev.

[30] Keniry A, Oxley D, Monnier P, Kyba M, Dandolo L, Smits G, Reik W (2012). The H19 lincRNA is a developmental reservoir of miR-675 that suppresses growth and Igf1r. Nat Cell Biol.

[31] Brunkow ME, Tilghman SM (1991). Ectopic expression of the H19 gene in mice causes prenatal lethality. Genes Dev.

[32] Dey BK, Pfeifer K, Dutta A (2014). The H19 long noncoding RNA gives rise to microRNAs miR-675-3p and miR-675-5p to promote skeletal muscle differentiation and regeneration. Genes Dev.

[33] Eun B, Sampley ML, Van Winkle MT, Good AL, Kachman MM, Pfeifer K (2013). The Igf2/H19 muscle enhancer is an active transcriptional complex. Nucleic Acids Res.

[34] Mouillet JF, Donker RB, Mishima T, Cronqvist T, Chu T, Sadovsky Y (2013). The unique expression and function of miR-424 in human placental trophoblasts. Biol Reprod.

[35] Huang L, Shen Z, Xu Q, Huang X, Chen Q, Li D (2013). Increased levels of microRNA-424 are associated with the pathogenesis of fetal growth restriction. Placenta.

[36] Fishilevich S, Nudel R, Rappaport N, Hadar R, Plaschkes I, Iny Stein T, Rosen N, Kohn A, Twik M, Safran M, Lancet D, Cohen D (2017). GeneHancer: genome-wide integration of enhancers and target genes in GeneCards. Database (Oxford).

[37] Jackman SM, Kong X, Fant ME (2012). Plac1 (placenta-specific 1) is essential for normal placental and embryonic development. Mol Reprod Dev.

[38] Grant J, Mahadevaiah SK, Khil P, Sangrithi MN, Royo H, Duckworth J, McCarrey JR, VandeBerg JL, Renfree MB, Taylor W, Elgar G, Camerini-Otero RD, Gilchrist MJ, Turner JM (2012). Rsx is a metatherian RNA with Xist-like properties in X-chromosome inactivation. Nature.

[39] Saga Y, Kitajima S, Miyagawa-Tomita S (2000). Mesp1 expression is the earliest sign of cardiovascular development. Trends Cardiovasc Med.

[40] Saga Y, Miyagawa-Tomita S, Takagi A, Kitajima S, Miyazaki J, Inoue T (1999). MesP1 is expressed in the heart precursor cells and required for the formation of a single heart tube. Development.

[41] Chan SS, Shi X, Toyama A, Arpke RW, Dandapat A, Iacovino M, Kang J, Le G, Hagen HR, Garry DJ, Kyba M (2013). Mesp1 patterns mesoderm into cardiac, hematopoietic, or skeletal myogenic progenitors in a context-dependent manner. Cell Stem Cell.

[42] Shen X, Soibam B, Benham A, Xu X, Chopra M, Peng X, Yu W, Bao W, Liang R, Azares A, Liu P, Gunaratne PH, Mercola M, Cooney AJ, Schwartz RJ, Liu Y (2016). miR-322/-503 cluster is expressed in the earliest cardiac progenitor cells and drives cardiomyocyte specification. Proc Natl Acad Sci USA.

[43] Sarkar S, Dey BK, Dutta A (2010). MiR-322/424 and -503 are induced during muscle differentiation and promote cell cycle quiescence and differentiation by down-regulation of Cdc25A. Mol Biol Cell.

[44] Hawke TJ, Meeson AP, Jiang N, Graham S, Hutcheson K, DiMaio JM, Garry DJ (2003). p21 is essential for normal myogenic progenitor cell function in regenerating skeletal muscle. Am J Physiol Cell Physiol.

[45] Myers TK, Andreuzza SE, Franklin DS (2004). p18INK4c and p27KIP1 are required for cell cycle arrest of differentiated myotubes. Exp Cell Res.

[46] Baskerville S, Bartel DP (2005). Microarray profiling of microRNAs reveals frequent coexpression with neighboring miRNAs and host genes. RNA.

[47] Sempere LF, Freemantle S, Pitha-Rowe I, Moss E, Dmitrovsky E, Ambros V (2004). Expression profiling of mammalian microRNAs uncovers a subset of brain-expressed microRNAs with possible roles in murine and human neuronal differentiation. Genome Biol.

[48] Wu H, Neilson JR, Kumar P, Manocha M, Shankar P, Sharp PA, Manjunath N (2007). miRNA profiling of naive, effector and memory CD8 T cells. PLoS One.

[49] Merkerova M, Belickova M, Bruchova H (2008). Differential expression of microRNAs in hematopoietic cell lineages. Eur J Haematol.

[50] Liu CG, Calin GA, Meloon B, Gamliel N, Sevignani C, Ferracin M, Dumitru CD, Shimizu M, Zupo S, Dono M, Alder H, Bullrich F, Negrini M, Croce CM (2004). An oligonucleotide microchip for genome-wide microRNA profiling in human and mouse tissues. Proc Natl Acad Sci USA.

[51] Rosa A, Ballarino M, Sorrentino A, Sthandier O, De Angelis FG, Marchioni M, Masella B, Guarini A, Fatica A, Peschle C, Bozzoni I (2007). The interplay between the master transcription factor PU1 and miR-424 regulates human monocyte/macrophage differentiation. Proc Natl Acad Sci USA.

[52] Forrest AR, Kanamori-Katayama M, Tomaru Y, Lassmann T, Ninomiya N, Takahashi Y, de Hoon MJ, Kubosaki A, Kaiho A, Suzuki M, Yasuda J, Kawai J, Hayashizaki Y, Hume DA, Suzuki H (2010). Induction of microRNAs, mir-155, mir-222, mir-424 and mir-503, promotes monocytic differentiation through combinatorial regulation. Leukemia.

[53] Hershkovitz-Rokah O, Modai S, Pasmanik-Chor M, Toren A, Shomron N, Raanani P, Shpilberg O, Granot G (2015). Restoration of miR-424 suppresses BCR-ABL activity and sensitizes CML cells to imatinib treatment. Cancer Lett.

[54] Pekarsky Y, Croce CM (2015). Role of miR-15/16 in CLL. Cell Death Differ.

[55] Pekarsky Y, Balatti V, Croce CM (2018). BCL2 and miR-15/16: from gene discovery to treatment. Cell Death Differ.

[56] Calin GA, Dumitru CD, Shimizu M, Bichi R, Zupo S, Noch E, Aldler H, Rattan S, Keating M, Rai K, Rassenti L, Kipps T, Negrini M, Bullrich F, Croce CM (2002). Frequent deletions and down-regulation of micro-RNA genes miR15 and miR16 at 13q14 in chronic lymphocytic leukemia. Proc Natl Acad Sci USA.

[57] Cimmino A, Calin GA, Fabbri M, Iorio MV, Ferracin M, Shimizu M, Wojcik SE, Aqeilan RI, Zupo S, Dono M, Rassenti L, Alder H, Volinia S, Liu CG, Kipps TJ, Negrini M, Croce CM (2005). miR-15 and miR-16 induce apoptosis by targeting BCL2. Proc Natl Acad Sci USA.

[58] Lerner M, Harada M, Loven J, Castro J, Davis Z, Oscier D, Henriksson M, Sangfelt O, Grander D, Corcoran MM (2009). DLEU2, frequently deleted in malignancy, functions as a critical host gene of the cell cycle inhibitory microRNAs miR-15a and miR-16-1. Exp Cell Res.

[59] Bandi N, Zbinden S, Gugger M, Arnold M, Kocher V, Hasan L, Kappeler A, Brunner T, Vassella E (2009). miR-15a and miR-16 are implicated in cell cycle regulation in a Rb-dependent manner and are frequently deleted or down-regulated in non-small cell lung cancer. Cancer Res.

[60] Chen RW, Bemis LT, Amato CM, Myint H, Tran H, Birks DK, Eckhardt SG, Robinson WA (2008). Truncation in CCND1 mRNA alters miR-16-1 regulation in mantle cell lymphoma. Blood.

[61] Bonifacio LN, Jarstfer MB (2010). MiRNA profile associated with replicative senescence, extended cell culture, and ectopic telomerase expression in human foreskin fibroblasts. PLoS One.

[62] Llobet-Navas D, Rodriguez-Barrueco R, Castro V, Ugalde AP, Sumazin P, Jacob-Sendler D, Demircan B, Castillo-Martin M, Putcha P, Marshall N, Villagrasa P, Chan J, Sanchez-Garcia F, Pe’er D, Rabadan R, Iavarone A, Cordon-Cardo C, Califano A, Lopez-Otin C, Ezhkova E, Silva JM (2014). The miR-424(322)/503 cluster orchestrates remodeling of the epithelium in the involuting mammary gland. Genes Dev.

[63] Rodriguez-Barrueco R, Nekritz EA, Bertucci F, Yu J, Sanchez-Garcia F, Zeleke TZ, Gorbatenko A, Birnbaum D, Ezhkova E, Cordon-Cardo C, Finetti P, Llobet-Navas D, Silva JM (2017). miR-424(322)/503 is a breast cancer tumor suppressor whose loss promotes resistance to chemotherapy. Genes Dev.

[64] Macias H, Hinck L (2012). Mammary gland development. Wiley Interdiscip Rev Dev Biol.

[65] Wang J, Wang S, Zhou J, Qian Q (2018). miR-424-5p regulates cell proliferation, migration and invasion by targeting doublecortin-like kinase 1 in basal-like breast cancer. Biomed Pharmacother.

[66] Gong J, Luk F, Jaiswal R, Bebawy M (2014). Microparticles mediate the intercellular regulation of microRNA-503 and proline-rich tyrosine kinase 2 to alter the migration and invasion capacity of breast cancer cells. Front Oncol.

[67] Zhou Y, An Q, Guo RX, Qiao YH, Li LX, Zhang XY, Zhao XL (2017). miR424-5p functions as an anti-oncogene in cervical cancer cell growth by targeting KDM5B via the Notch signaling pathway. Life Sci.

[68] Xu J, Li Y, Wang F, Wang X, Cheng B, Ye F, Xie X, Zhou C, Lu W (2013). Suppressed miR-424 expression via upregulation of target gene Chk1 contributes to the progression of cervical cancer. Oncogene.

[69] Jiang L, Zhao Z, Zheng L, Xue L, Zhan Q, Song Y (2017). Downregulation of miR-503 promotes ESCC cell proliferation, migration, and invasion by targeting cyclin D1. Genom Proteom Bioinform.

[70] Luo J, Wang Z, Huang J, Yao Y, Sun Q, Wang J, Shen Y (2018). HOXC13 promotes proliferation of esophageal squamous cell carcinoma via repressing transcription of CASP3. Cancer Sci.

[71] Wu J, Gao F, Xu T, Deng X, Wang C, Yang X, Hu Z, Long Y, He X, Liang G, Ren D, Dai T (2018). miR-503 suppresses the proliferation and metastasis of esophageal squamous cell carcinoma by triggering autophagy via PKA/mTOR signaling. Int J Oncol.

[72] Zhang Y, Li T, Guo P, Kang J, Wei Q, Jia X, Zhao W, Huai W, Qiu Y, Sun L, Han L (2014). MiR-424-5p reversed epithelial-mesenchymal transition of anchorage-independent HCC cells by directly targeting ICAT and suppressed HCC progression. Sci Rep.

[73] Zhou B, Ma R, Si W, Li S, Xu Y, Tu X, Wang Q (2013). MicroRNA-503 targets FGF2 and VEGFA and inhibits tumor angiogenesis and growth. Cancer Lett.

[74] Yang X, Zang J, Pan X, Yin J, Xiang Q, Yu J, Gan R, Lei X (2017). miR-503 inhibits proliferation making human hepatocellular carcinoma cells susceptible to 5-fluorouracil by targeting EIF4E. Oncol Rep.

[75] Xiao F, Zhang W, Chen L, Chen F, Xie H, Xing C, Yu X, Ding S, Chen K, Guo H, Cheng J, Zheng S, Zhou L (2013). MicroRNA-503 inhibits the G1/S transition by downregulating cyclin D3 and E2F3 in hepatocellular carcinoma. J Transl Med.

[76] Yang Y, Liu L, Zhang Y, Guan H, Wu J, Zhu X, Yuan J, Li M (2014). MiR-503 targets PI3K p85 and IKK-beta and suppresses progression of non-small cell lung cancer. Int J Cancer.

[77] Xu S, Tao Z, Hai B, Liang H, Shi Y, Wang T, Song W, Chen Y, OuYang J, Chen J, Kong F, Dong Y, Jiang SW, Li W, Wang P, Yuan Z, Wan X, Wang C, Li W, Zhang X, Chen K (2016). miR-424(322) reverses chemoresistance via T-cell immune response activation by blocking the PD-L1 immune checkpoint. Nat Commun.

[78] Wu X, Ruan Y, Jiang H, Xu C (2017). MicroRNA-424 inhibits cell migration, invasion, and epithelial mesenchymal transition by downregulating doublecortin-like kinase 1 in ovarian clear cell carcinoma. Int J Biochem Cell Biol.

[79] Jiang X, Chen Y, Du E, Yang K, Zhang Z, Qi S, Xu Y (2016). GATA3-driven expression of miR-503 inhibits prostate cancer progression by repressing ZNF217 expression. Cell Signal.

[80] Li Q, Qiu XM, Li QH, Wang XY, Li L, Xu M, Dong M, Xiao YB (2015). MicroRNA-424 may function as a tumor suppressor in endometrial carcinoma cells by targeting E2F7. Oncol Rep.

[81] Xu YY, Wu HJ, Ma HD, Xu LP, Huo Y, Yin LR (2013). MicroRNA-503 suppresses proliferation and cell-cycle progression of endometrioid endometrial cancer by negatively regulating cyclin D1. FEBS J.

[82] Varghese VK, Shukla V, Kabekkodu SP, Pandey D, Satyamoorthy K (2018). DNA methylation regulated microRNAs in human cervical cancer. Mol Carcinog.

[83] Hong S, Cheng S, Songock W, Bodily J, Laimins LA (2017). Suppression of microRNA 424 levels by human papillomaviruses is necessary for differentiation-dependent genome amplification. J Virol.

[84] Tian Q, Li Y, Wang F, Li Y, Xu J, Shen Y, Ye F, Wang X, Cheng X, Chen Y, Wan X, Lu W, Xie X (2014). MicroRNA detection in cervical exfoliated cells as a triage for human papillomavirus-positive women. J Natl Cancer Inst.

[85] Cheung TH, Man KN, Yu MY, Yim SF, Siu NS, Lo KW, Doran G, Wong RR, Wang VW, Smith DI, Worley MJ, Berkowitz RS, Chung TK, Wong YF (2012). Dysregulated microRNAs in the pathogenesis and progression of cervical neoplasm. Cell Cycle.

[86] Liu J, Gu Z, Tang Y, Hao J, Zhang C, Yang X (2018). Tumour-suppressive microRNA-424-5p directly targets CCNE1 as potential prognostic markers in epithelial ovarian cancer. Cell Cycle.

[87] Wei SC, Duffy CR, Allison JP (2018). Fundamental mechanisms of immune checkpoint blockade therapy. Cancer Discov.

[88] Haikalis ME, Wessels JM, Leyland NA, Agarwal SK, Foster WG (2018). MicroRNA expression pattern differs depending on endometriosis lesion type. Biol Reprod.

[89] Braza-Boils A, Mari-Alexandre J, Gilabert J, Sanchez-Izquierdo D, Espana F, Estelles A, Gilabert-Estelles J (2014). MicroRNA expression profile in endometriosis: its relation to angiogenesis and fibrinolytic factors. Hum Reprod.

[90] Ohlsson Teague EM, Van der Hoek KH, Van der Hoek MB, Perry N, Wagaarachchi P, Robertson SA, Print CG, Hull LM (2009). MicroRNA-regulated pathways associated with endometriosis. Mol Endocrinol.

[91] Pande HO, Tesfaye D, Hoelker M, Gebremedhn S, Held E, Neuhoff C, Tholen E, Schellander K, Wondim DS (2018). MicroRNA-424/503 cluster members regulate bovine granulosa cell proliferation and cell cycle progression by targeting SMAD7 gene through activin signalling pathway. J Ovarian Res.

[92] Gebremedhn S, Salilew-Wondim D, Ahmad I, Sahadevan S, Hossain MM, Hoelker M, Rings F, Neuhoff C, Tholen E, Looft C, Schellander K, Tesfaye D (2015). MicroRNA expression profile in bovine granulosa cells of preovulatory dominant and subordinate follicles during the late follicular phase of the estrous cycle. PLoS One.

[93] Salilew-Wondim D, Ahmad I, Gebremedhn S, Sahadevan S, Hossain MD, Rings F, Hoelker M, Tholen E, Neuhoff C, Looft C, Schellander K, Tesfaye D (2014). The expression pattern of microRNAs in granulosa cells of subordinate and dominant follicles during the early luteal phase of the bovine estrous cycle. PLoS One.

[94] Moreno JM, Nunez MJ, Quinonero A, Martinez S, de la Orden M, Simon C, Pellicer A, Diaz-Garcia C, Dominguez F (2015). Follicular fluid and mural granulosa cells microRNA profiles vary in in vitro fertilization patients depending on their age and oocyte maturation stage. Fertil Steril.

[95] Baran-Gale J, Purvis JE, Sethupathy P (2016). An integrative transcriptomics approach identifies miR-503 as a candidate master regulator of the estrogen response in MCF-7 breast cancer cells. RNA.

[96] Cicatiello L, Mutarelli M, Grober OM, Paris O, Ferraro L, Ravo M, Tarallo R, Luo S, Schroth GP, Seifert M, Zinser C, Chiusano ML, Traini A, De Bortoli M, Weisz A (2010). Estrogen receptor alpha controls a gene network in luminal-like breast cancer cells comprising multiple transcription factors and microRNAs. Am J Pathol.

[97] Connolly M, Paul R, Farre-Garros R, Natanek SA, Bloch S, Lee J, Lorenzo JP, Patel H, Cooper C, Sayer AA, Wort SJ, Griffiths M, Polkey MI, Kemp PR (2017). miR-424-5p reduces ribosomal RNA and protein synthesis in muscle wasting. J Cachexia Sarcopenia Muscle.

[98] Morikawa M, Derynck R, Miyazono K (2016). TGF-beta and the TGF-beta family: context-dependent roles in cell and tissue physiology. Cold Spring Harb Perspect Biol.

[99] Weiss A, Attisano L (2013). The TGFbeta superfamily signaling pathway. Wiley Interdiscip Rev Dev Biol.

[100] Massague J (2012). TGFbeta signalling in context. Nat Rev Mol Cell Biol.

[101] Guo P, Yu Y, Li H, Zhang D, Gong A, Li S, Liu W, Cheng L, Qiu Y, Yao W, Li L, Feng Y (2017). TGF-a1-induced miR-503 controls cell growth and apoptosis by targeting PDCD4 in glioblastoma cells. Sci Rep.

[102] Gu W, Hong X, Le Bras A, Nowak WN, Issa Bhaloo S, Deng J, Xie Y, Hu Y, Ruan XZ, Xu Q (2018). Smooth muscle cells differentiated from mesenchymal stem cells are regulated by microRNAs and suitable for vascular tissue grafts. J Biol Chem.

[103] Xiao X, Huang C, Zhao C, Gou X, Senavirathna LK, Hinsdale M, Lloyd P, Liu L (2015). Regulation of myofibroblast differentiation by miR-424 during epithelial-to-mesenchymal transition. Arch Biochem Biophys.

[104] Zhang D, Wang Y, Shi Z, Liu J, Sun P, Hou X, Zhang J, Zhao S, Zhou BP, Mi J (2015). Metabolic reprogramming of cancer-associated fibroblasts by IDH3alpha downregulation. Cell Rep.

[105] Sun Y, Xu J, Xu L, Zhang J, Chan K, Pan X, Li G (2017). MiR-503 promotes bone formation in distraction osteogenesis through suppressing Smurf1 expression. Sci Rep.

[106] Cao S, Xiao L, Rao JN, Zou T, Liu L, Zhang D, Turner DJ, Gorospe M, Wang JY (2014). Inhibition of Smurf2 translation by miR-322/503 modulates TGF-beta/Smad2 signaling and intestinal epithelial homeostasis. Mol Biol Cell.

[107] Drasin DJ, Guarnieri AL, Neelakantan D, Kim J, Cabrera JH, Wang CA, Zaberezhnyy V, Gasparini P, Cascione L, Huebner K, Tan AC, Ford HL (2015). TWIST1-induced miR-424 reversibly drives mesenchymal programming while inhibiting tumor initiation. Cancer Res.

[108] Li D, Liu K, Li Z, Wang J, Wang X (2017). miR-19a and miR-424 target TGFBR3 to promote epithelial-to-mesenchymal transition and migration of tongue squamous cell carcinoma cells. Cell Adhes Migr.

[109] Banyard J, Chung I, Wilson AM, Vetter G, Le Bechec A, Bielenberg DR, Zetter BR (2013). Regulation of epithelial plasticity by miR-424 and miR-200 in a new prostate cancer metastasis model. Sci Rep.

[110] Torres S, Garcia-Palmero I, Bartolome RA, Fernandez-Acenero MJ, Molina E, Calvino E, Segura MF, Casal JI (2017). Combined miRNA profiling and proteomics demonstrates that different miRNAs target a common set of proteins to promote colorectal cancer metastasis. J Pathol.

[111] Thomas SJ, Snowden JA, Zeidler MP, Danson SJ (2015). The role of JAK/STAT signalling in the pathogenesis, prognosis and treatment of solid tumours. Br J Cancer.

[112] Peng HY, Jiang SS, Hsiao JR, Hsiao M, Hsu YM, Wu GH, Chang WM, Chang JY, Jin SL, Shiah SG (2016). IL-8 induces miR-424-5p expression and modulates SOCS2/STAT5 signaling pathway in oral squamous cell carcinoma. Mol Oncol.

[113] Dallavalle C, Albino D, Civenni G, Merulla J, Ostano P, Mello-Grand M, Rossi S, Losa M, D’Ambrosio G, Sessa F, Thalmann GN, Garcia-Escudero R, Zitella A, Chiorino G, Catapano CV, Carbone GM (2016). MicroRNA-424 impairs ubiquitination to activate STAT3 and promote prostate tumor progression. J Clin Investig.

[114] Zhang J, Liu H, Hou L, Wang G, Zhang R, Huang Y, Chen X, Zhu J (2017). Circular RNA_LARP4 inhibits cell proliferation and invasion of gastric cancer by sponging miR-424-5p and regulating LATS1 expression. Mol Cancer.

[115] Mizrahi A, Barzilai A, Gur-Wahnon D, Ben-Dov IZ, Glassberg S, Meningher T, Elharar E, Masalha M, Jacob-Hirsch J, Tabibian-Keissar H, Barshack I, Roszik J, Leibowitz-Amit R, Sidi Y, Avni D (2018). Alterations of microRNAs throughout the malignant evolution of cutaneous squamous cell carcinoma: the role of miR-497 in epithelial to mesenchymal transition of keratinocytes. Oncogene.

[116] Babapoor S, Wu R, Kozubek J, Auidi D, Grant-Kels JM, Dadras SS (2017). Identification of microRNAs associated with invasive and aggressive phenotype in cutaneous melanoma by next-generation sequencing. Lab Investig.

[117] Majmundar AJ, Wong WJ, Simon MC (2010). Hypoxia-inducible factors and the response to hypoxic stress. Mol Cell.

[118] Ghosh G, Subramanian IV, Adhikari N, Zhang X, Joshi HP, Basi D, Chandrashekhar YS, Hall JL, Roy S, Zeng Y, Ramakrishnan S (2010). Hypoxia-induced microRNA-424 expression in human endothelial cells regulates HIF-alpha isoforms and promotes angiogenesis. J Clin Investig.

[119] Zhang D, Shi Z, Li M, Mi J (2014). Hypoxia-induced miR-424 decreases tumor sensitivity to chemotherapy by inhibiting apoptosis. Cell Death Dis.

[120] Liu P, Zhao H, Wang R, Wang P, Tao Z, Gao L, Yan F, Liu X, Yu S, Ji X, Luo Y (2015). MicroRNA-424 protects against focal cerebral ischemia and reperfusion injury in mice by suppressing oxidative stress. Stroke.

[121] Walter P, Ron D (2011). The unfolded protein response: from stress pathway to homeostatic regulation. Science.

[122] Gupta A, Hossain MM, Read DE, Hetz C, Samali A, Gupta S (2015). PERK regulated miR-424(322)-503 cluster fine-tunes activation of IRE1 and ATF6 during unfolded protein response. Sci Rep.

[123] Sudarshan S, Sourbier C, Kong HS, Block K, Valera Romero VA, Yang Y, Galindo C, Mollapour M, Scroggins B, Goode N, Lee MJ, Gourlay CW, Trepel J, Linehan WM, Neckers L (2009). Fumarate hydratase deficiency in renal cancer induces glycolytic addiction and hypoxia-inducible transcription factor 1alpha stabilization by glucose-dependent generation of reactive oxygen species. Mol Cell Biol.

[124] Klimova T, Chandel NS (2008). Mitochondrial complex III regulates hypoxic activation of HIF. Cell Death Differ.

[125] Kaelin WG, Ratcliffe PJ (2008). Oxygen sensing by metazoans: the central role of the HIF hydroxylase pathway. Mol Cell.

[126] Nandy SB, Orozco A, Lopez-Valdez R, Roberts R, Subramani R, Arumugam A, Dwivedi AK, Stewart V, Prabhakar G, Jones S, Lakshmanaswamy R (2017). Glucose insult elicits hyperactivation of cancer stem cells through miR-424–cdc42–prdm14 signalling axis. Br J Cancer.

[127] Wang G, Gu Y, Xu N, Zhang M, Yang T (2018). Decreased expression of miR-150, miR146a and miR424 in type 1 diabetic patients: association with ongoing islet autoimmunity. Biochem Biophys Res Commun.

[128] Reid G, Kirschner MB, van Zandwijk N (2011). Circulating microRNAs: association with disease and potential use as biomarkers. Crit Rev Oncol Hematol.

[129] Creemers EE, Tijsen AJ, Pinto YM (2012). Circulating microRNAs: novel biomarkers and extracellular communicators in cardiovascular disease?. Circ Res.

[130] Valadi H, Ekstrom K, Bossios A, Sjostrand M, Lee JJ, Lotvall JO (2007). Exosome-mediated transfer of mRNAs and microRNAs is a novel mechanism of genetic exchange between cells. Nat Cell Biol.

[131] Skog J, Wurdinger T, van Rijn S, Meijer DH, Gainche L, Sena-Esteves M, Curry WT, Carter BS, Krichevsky AM, Breakefield XO (2008). Glioblastoma microvesicles transport RNA and proteins that promote tumour growth and provide diagnostic biomarkers. Nat Cell Biol.

[132] Ivo D’Urso P, Fernando D’Urso O, Damiano Gianfreda C, Mezzolla V, Storelli C, Marsigliante S (2015). miR-15b and miR-21 as circulating biomarkers for diagnosis of glioma. Curr Genom.

[133] Chiam K, Wang T, Watson DI, Mayne GC, Irvine TS, Bright T, Smith L, White IA, Bowen JM, Keefe D, Thompson SK, Jones ME, Hussey DJ (2015). Circulating serum exosomal miRNAs as potential biomarkers for esophageal adenocarcinoma. J Gastrointest Surg.

[134] Zhang Y, Zhang D, Wang F, Xu D, Guo Y, Cui W (2015). Serum miRNAs panel (miR-16-2*, miR-195, miR-2861, miR-497) as novel non-invasive biomarkers for detection of cervical cancer. Sci Rep.

[135] Shin VY, Siu JM, Cheuk I, Ng EK, Kwong A (2015). Circulating cell-free miRNAs as biomarker for triple-negative breast cancer. Br J Cancer.

[136] Guo S, Guo W, Li S, Dai W, Zhang N, Zhao T, Wang H, Ma J, Yi X, Ge R, Wang G, Gao T, Li C (2016). Serum miR-16: a potential biomarker for predicting melanoma prognosis. J Investig Dermatol.

[137] Vegter EL, Schmitter D, Hagemeijer Y, Ovchinnikova ES, van der Harst P, Teerlink JR, O’Connor CM, Metra M, Davison BA, Bloomfield D, Cotter G, Cleland JG, Givertz MM, Ponikowski P, van Veldhuisen DJ, van der Meer P, Berezikov E, Voors AA, Khan MA (2016). Use of biomarkers to establish potential role and function of circulating microRNAs in acute heart failure. Int J Cardiol.

[138] Bye A, Rosjo H, Nauman J, Silva GJ, Follestad T, Omland T, Wisloff U (2016). Circulating microRNAs predict future fatal myocardial infarction in healthy individuals—the HUNT study. J Mol Cell Cardiol.

[139] de Andrade HM, de Albuquerque M, Avansini SH, de Rocha CD, Dogini DB, Nucci A, Carvalho B, Lopes-Cendes I, Franca MC (2016). MicroRNAs-424 and 206 are potential prognostic markers in spinal onset amyotrophic lateral sclerosis. J Neurol Sci.

[140] Berghmans T, Ameye L, Willems L, Paesmans M, Mascaux C, Lafitte JJ, Meert AP, Scherpereel A, Cortot AB, Cstoth I, Dernies T, Toussaint L, Leclercq N, Sculier JP, European Lung Cancer Working P (2013). Identification of microRNA-based signatures for response and survival for non-small cell lung cancer treated with cisplatin–vinorelbine A ELCWP prospective study. Lung Cancer.

[141] Joerger M, Baty F, Fruh M, Droege C, Stahel RA, Betticher DC, von Moos R, Ochsenbein A, Pless M, Gautschi O, Rothschild S, Brauchli P, Klingbiel D, Zappa F, Brutsche M (2014). Circulating microRNA profiling in patients with advanced non-squamous NSCLC receiving bevacizumab/erlotinib followed by platinum-based chemotherapy at progression (SAKK 19/05). Lung Cancer.

[142] Zhang L, Xu Y, Jin X, Wang Z, Wu Y, Zhao D, Chen G, Li D, Wang X, Cao H, Xie Y, Liang Z (2015). A circulating miRNA signature as a diagnostic biomarker for non-invasive early detection of breast cancer. Breast Cancer Res Treat.

[143] Alvarez-Mora MI, Rodriguez-Revenga L, Madrigal I, Torres-Silva F, Mateu-Huertas E, Lizano E, Friedlander MR, Marti E, Estivill X, Mila M (2013). MicroRNA expression profiling in blood from fragile X-associated tremor/ataxia syndrome patients. Genes Brain Behav.

[144] Anastasilakis AD, Yavropoulou MP, Makras P, Sakellariou GT, Papadopoulou F, Gerou S, Papapoulos SE (2017). Increased osteoclastogenesis in patients with vertebral fractures following discontinuation of denosumab treatment. Eur J Endocrinol.

[145] Chen C, Cheng P, Xie H, Zhou HD, Wu XP, Liao EY, Luo XH (2014). MiR-503 regulates osteoclastogenesis via targeting RANK. J Bone Miner Res Off J Am Soc Bone Miner Res.

[146] Baptista R, Marques C, Catarino S, Enguita FJ, Costa MC, Matafome P, Zuzarte M, Castro G, Reis A, Monteiro P, Pego M, Pereira P, Girao H (2018). MicroRNA-424(322) as a new marker of disease progression in pulmonary arterial hypertension and its role in right ventricular hypertrophy by targeting SMURF1. Cardiovasc Res.

[147] Snowhite IV, Allende G, Sosenko J, Pastori RL, Messinger Cayetano S, Pugliese A (2017). Association of serum microRNAs with islet autoimmunity, disease progression and metabolic impairment in relatives at risk of type 1 diabetes. Diabetologia.

[148] Wang X, Sundquist K, Elf JL, Strandberg K, Svensson PJ, Hedelius A, Palmer K, Memon AA, Sundquist J, Zoller B (2016). Diagnostic potential of plasma microRNA signatures in patients with deep-vein thrombosis. Thromb Haemost.

[149] Marques FZ, Vizi D, Khammy O, Mariani JA, Kaye DM (2016). The transcardiac gradient of cardio-microRNAs in the failing heart. Eur J Heart Fail.

[150] Fei Y, Hou J, Xuan W, Zhang C, Meng X (2018). The relationship of plasma miR-503 and coronary collateral circulation in patients with coronary artery disease. Life Sci.

[151] de Gonzalo-Calvo D, Davalos A, Montero A, Garcia-Gonzalez A, Tyshkovska I, Gonzalez-Medina A, Soares SM, Martinez-Camblor P, Casas-Agustench P, Rabadan M, Diaz-Martinez AE, Ubeda N, Iglesias-Gutierrez E (2015). Circulating inflammatory miRNA signature in response to different doses of aerobic exercise. J Appl Physiol.

